# Supplementary material for: Preparation of W/O Hypaphorine–Chitosan Nanoparticles and Its Application on Promoting Chronic Wound Healing via Alleviating Inflammation Block
Source: Nanomaterials (Basel). 2021 Oct 25;11(11):2830. doi: 10.3390/nano11112830 (PMC8625710; doi:10.3390/nano11112830)
Supplement: Supplementary file 1 [file nanomaterials-11-02830-s001.zip › nanomaterials-1401735-supplementary.pdf]

Main components of Hua Tuo Lithospermum burn ointment

The main components of Hua Tuo Lithospermum burn ointment（HT） are Lithospermum, borneol, Coptis, Sophora flavescens, lanolin, Phellodendron chinense, allantoin and vaseline.
